# Factors associated with alcohol use disorder: the role of depression, anxiety, stress, alexithymia and work fatigue- a population study in Lebanon

**DOI:** 10.1186/s12889-020-8345-1

**Published:** 2020-02-18

**Authors:** Sahar Obeid, Marwan Akel, Chadia Haddad, Kassandra Fares, Hala Sacre, Pascale Salameh, Souheil Hallit

**Affiliations:** 1 Research and Psychology departments, Psychiatric Hospital of the Cross, Jal Eddib, Lebanon; 2 Faculty of Arts and Sciences, Holy Spirit University of Kaslik (USEK), Jounieh, Lebanon; 3 INSPECT-LB: Institut National de Santé Publique, Epidémiologie Clinique et Toxicologie – Liban, Beirut, Lebanon; 4 School of Pharmacy, Lebanese International University, Beirut, Lebanon; 5 INSERM, U1094, Tropical Neuroepidemiology, Limoges, France; 6 Univ. Limoges, UMR 1094, Tropical Neuroepidemiology, Institute of Epidemiology and Tropical Neurology, GEIST, 87000 Limoges, France; 7 Department of psychiatry, CH Esquirol, 87025 Limoges, France; 8 Drug Information Center, Order of Pharmacists of Lebanon, Beirut, Lebanon; 9 Faculty of Pharmacy, Lebanese University, Beirut, Lebanon; 10 Faculty of Medicine, Lebanese University, Beirut, Lebanon; 11 Faculty of Medicine and Medical Sciences, Holy Spirit University of Kaslik (USEK), Jounieh, Lebanon

**Keywords:** Alcohol use disorder, Depression, Anxiety, Alexithymia, Self-esteem

## Abstract

**Background:**

International research showed that common mental disorders such as depression, anxiety, social anxiety, stress, alexithymia and having insecure attachment styles are risk factors for alcohol use disorder (AUD). Our objective was to study the factors associated withAUD in a sample of the Lebanese population.

**Methods:**

During the period lasting from November 2017 to March 2018, a sample of 789 Lebanese participants agreed to contribute to a cross-sectional study (53.23% males). Alcohol use disorder was assessed using the Alcohol Use Disorder Identification Test (AUDIT).

**Results:**

A high risk of AUD was associated with higher alexithymia (ORa = 1.030; CI 1.009–1.051), depression (ORa = 1.076; CI 1.050–1.103) and suicidal ideation (ORa = 1.253; CI 1.026–1.531) in a significant manner. In opposition, a higher number of kids (ORa = 0.863; CI 0.752–0.991), being a female (ORa = 0.460; CI 0.305–0.694) and higher emotional management (ORa = 0.962; CI 0.937–0.988) were significantly associated with lower AUD risk.

A cluster analysis derived three mutually exclusive clusters. Cluster 1 formed 45.4% of the sample and assembled people with psychological difficulties (work fatigue and high stress, high emotional work fatigue and low emotional intelligence, low self-esteem, high social phobia, high alexithymia); Cluster 2 formed 34.4% of the sample and assembled people with high wellbeing (low suicidal ideation, low emotional work fatigue, depression and anxiety, high emotional intelligence, high self-esteem and low social phobia); whereas cluster 3 formed 20.2% of the sample and represented people with mental dysfunction (high anxiety and depression, high suicidal ideation, low self-esteem and high social phobia, low emotional intelligence, high emotional work fatigue). People with psychological difficulties (cluster 1) (Beta = 5.547; CI 4.430–6.663), and people in distress (cluster 3) (Beta = 7.455; CI 5.945–8.965) were associated with higher AUDIT scores than those with high wellbeing (cluster 2).

**Conclusion:**

AUD seems to be influenced by several factors among the Lebanese population, including alexithymia, stress, anxiety and work fatigue. Healthcare professionals should spread awareness to reduce the prevalence of these factors.

## Background

Alcoholism, also known as alcohol use disorder (AUD), is a broad term for any drinking of alcohol that results in mental or physical health problems [1]. Alcohol use disorder is the third leading risk factor for the global burden of disease [2]. The prevalence of alcohol use disorder varies around the world, being highest in Northern and Eastern Europe [3] and parts of the Americas (8.4% in adult men and 4.2% in adult women) [4], while the ranges observed in the Mediterranean countries vary between 1.7% [5] and 11.2% [6]. A study carried out in Lebanon showed that the 12-month alcoholism prevalence is 6.2% [7]. However, these numbers are expected to be an underestimation since alcohol consumption is prohibited by Islam and may be clandestine among Muslims [8].

In particular, studies in Lebanon have shown that alcohol consumption is a major public health problem among adolescents and young adults. Thus, a 40% increase in the alcohol consumption was seen among elementary students between 2005 and 2011, with 85% having drunk their first glass of alcohol before the age of 14 [9]. In addition, young Lebanese drink more frequently than occasionally; 40% of high school students and half of university students consume alcohol at least 1 to 2 days per week [10, 11]. This is explained by the ease of access to alcohol [9]. However, the availability of alcohol in restaurants, cafes, bars, or pubs depends on the licensing of the on-site outlets according to the Lebanese laws and regulations (Decision no. 3210, issued in 1974). Also, policies regulating the quantities of alcohol-containing products in supermarkets and other stores are also lacking in the country [12].

Alcohol consumption is the result of different factors including economic, environmental, cultural, biological, social and psychological aspects that interact together and affect the propensity for a human to use this substance. In fact, alcohol experience is an interaction between alcohol itself, the user and the surroundings [13]. Moreover, research has shown that patients with common mental disorders such as depression, anxiety, social anxiety, stress, difficulty in expressing emotions are at higher risk of alcohol use disorder [14]. Further, a high prevalence of insecure attachment styles (anxious-ambivalent and avoidant styles) was demonstrated among alcohol addicted inpatients compared to normative samples [15]. In addition, previous research has demonstrated the effect of stress and work fatigue on high alcohol consumption [16]. Alcohol use disorder could be considered an avoidance emotion and a coping strategy to alleviate stress [17]. Family risk for alcohol use disorder, low social and personal resources for coping with stress, and positive expectations of alcohol effects [18] are associated with individual vulnerability to alcohol use disorder. Furthermore, alcohol and alexithymia are highly related since alexithymic individuals do not feel comfortable at public events and would abuse alcohol to improve interpersonal behavior [19].

We realized that no studies have been conducted in Lebanon on factors associated with AUD. Therefore, the primary objective of our study was to assess independent factors associated with alcohol use disorder among a representative sample of the Lebanese population. Secondary objectives were to evaluate the association between groups of factors and clusters associated with AUD in the same sample. The results will allow us to compare facts from Lebanon with those obtained in Western countries.

## Methods

### Sampling and data collection

Participants were 789 community residents who agreed to enroll in this cross sectional study between January and December 2018, using a proportionate sample from the five Mohafazat in the country. The latter are divided into Caza (stratum), then villages. From a list provided by the Central Agency of Statistics in Lebanon, we chose two villages per Caza where the questionnaire was distributed randomly to the households, based on a random sampling technique to select the included house [20]. Questionnaires were completed via a face-to-face interview by clinical psychologists who were independent from the study. All individuals over the age of 18 were eligible to participate. Excluded were those with mental illness (schizophrenia and bipolar disorder) or dementia (as reported by a family member). The methodology used has been previously described elsewhere [21–41].

### Minimal sample size calculation

According to Epi-info software, a minimal sample of 180 persons was needed based on a frequency of 6.2% of AUD among the general population according to previous findings [7], a 5% error and a design effect of 2.

### Questionnaire

The questionnaire was in Arabic. In the first part, questions concerned the sociodemographic characteristics of each individual: age, gender, socioeconomic status (SES), marital status, education level and type of alcohol drunk. Then, the questionnaire included numerous validated scales that served our study purpose, which were also used in a previous paper as follows:

### The alcohol use disorders identification test (AUDIT)

This test, composed of 10 items, is a tool to evaluate the use of alcohol, patterns when drinking alcohol, and problems alcohol-linked [42], which can be administered by a clinician or self-administered. Scores of 8 or above indicated hazardous/harmful alcohol use (high risk AUD), whereas scores below 8 indicated low AUD risk (Cronbach alpha = 0.885).

### Toronto alexithymia scale (TAS-20)

In order to evaluate alexithymia, we used the TAS-20 scale [43], with the questions answers graded based on a five-point Likert scale (1 = strongly disagree to 5 = strongly agree). Higher scores indicated higher alexithymia (Cronbach alpha = 0.778).

### Rosenberg self-esteem scale (RSES)

Both negative and positive feelings of any individual can be assessed by the RSES 10 item scale [44]. The answers of the questions are graded based on a four-point Likert scale with 1 or strongly agree to 4 or strongly disagree. Higher scores are signs of greater self-esteem (Cronbach alpha = 0.733).

### Hamilton depression rating scale (HDRS)

A validated Arabic version of the HDRS was used [45]. The first 17 items of the HDRS are scored and measure the severity of depressive symptoms [46]. Higher scores indicated higher depression (Cronbach alpha = 0.890).

### Hamilton anxiety scale (HAM-A)

A validated version of the HAM-A was used [47, 48]. It is formed of fourteen items, ranged according to a four-point Likert scale with zero or no symptoms to four or very severe symptoms. Greater score values indicated higher levels of anxiety (Cronbach alpha = 0.898).

### Evaluation of the three-dimensional work fatigue inventory (3D-WFI)

Work-associated physical, mental and emotional fatigue are evaluated respectively using the 18-item 3D-WFI scale [49]. Questions’ scoring ranked from 0 or never to 4 or every day. For all three studied factors, higher scores reflect greater levels of fatigue (Cronbach alpha for work fatigue were 0.823, 0.667 and 0.909 respectively).

### Columbia–suicide severity rating scale (C-SSRS)

It is an assessment instrument that measures suicidal behavior and ideation. As for the scoring, 0 means that the ideation of suicide is absent, whereas scoring 1 or above indicates a possible presence of suicidal ideation [50] (Cronbach alpha = 0.762).

### The perceived stress scale (PSS)

This scale is composed of ten items, which measures stress in the last month, with greater scores pointing out higher perceived stress (Cronbach alpha = 0.667).

### Liebowitz social anxiety scale (LSAS)

This scale features 24 items to assess performance anxiety and social situations based on a Likert scale from 0 to 3. Higher scores reflect higher social fear and avoidance. The Cronbach’s alpha values were for total score 0.954, for fear subscale 0.945 and for avoidance subscale 0.953.

### The quick emotional intelligence self-assessment

This scale assesses four subscales, emotional alertness, emotional control, social emotional awareness and relationship management. In all four domains, higher scores show higher emotional intelligence [51]. The Cronbach’s alpha values for the above mentioned subscales were 0.823, 0.888, 0.902, 0.908 respectively.

The questionnaire scales underwent both forward and back translations. At first, a mental health specialist translated all scales from English to Arabic. Then another mental health expert performed a translation from Arabic back to English. No significant differences were found between both English versions; therefore, the Arabic questionnaire was used as is.

### Statistical analyses

The statistical data analysis was conducted using the 23rd version of the SPSS software. When comparing two means we used the independent-sample t-test, whereas the analysis of variance (ANOVA) was used to compare three or more means. Bonferroni adjustment was used for ANOVA post hoc tests of between groups comparison. Chi-2 was used for categorical variables’ comparisons.

As for the reduction of data, factor and cluster analyses were used. Factor analysis was executed to detect groups of risk factors that go together and are associated with AUD in the sample. The latter adequacy with Bartlett’s Chi-square test of sphericity and Kaiser–Meyer–Olkin (KMO) index was confirmed as a preliminary step. Factors were extracted using a principal component analysis method and a promax rotation since factors were correlated. In this study, we retained factors with an Eigenvalue > 1. Items with factor loading values > 0.4 were taken as loading on a factor. Reliability was assessed using Cronbach’s alpha values. Second, we executed a cluster analysis with the identified factor scores to reflect profiles of participants, using the K-mean method. Analysis allowed for 10 iterations centering results on zero and convergence was only reached using a three-cluster structure, i.e., three different profiles.

Finally, several multivariable analyses were conducted using variables, factors and profiles: A backward logistic regression was conducted taking the dichotomous alcohol use disorder variable (low vs. high risk) as the dependent variable. Three stepwise linear regressions were conducted, taking the AUDIT result as the dependent variable. All variables/factors/clusters that showed a *p* < 0.1 in the bivariate analysis were taken as independent variables in the model, including sociodemographic and psychological variables. In all cases, a *p* < 0.05 was considered significant.

## Results

Of the 950 questionnaires distributed, 789 (83.05%) were completed and collected. A significantly higher proportion of male participants compared to females (58.8 vs 38.1%; *p* < 0.001), with a primary level of education compared to all other categories (p < 0.001) and widowed compared to all other marital statuses (*p* = 0.001) had high risk AUD. A significantly lower mean of number of kids was found in participants at high risk of AUD compared to low risk participants (0.62 vs. 0.91; *p* = 0.008) (Table 1). The post-hoc analysis results showed that a significantly higher percentage of participants with a primary level of education had high risk AUD compared to those with secondary (76.9% vs 48.2%; *p* = 0.002), university (76.9% vs 44.2%; *p* < 0.001) and higher university (76.9% vs 43.8%) levels of education. Moreover, a significantly higher percentage of participants with a complementary education level had high risk AUD compared to those with secondary (65.4% vs 48.2%l *p* = 0.04), university (65.4% vs 44.2%; *p* = 0.004) and higher university (65.4% vs 43.8%; *p* = 0.02) education levels. Finally, a significantly higher percentage of widowed (84.2% vs 47.1%; *p* = 0.002) and divorced (73.3% vs 47.1%; *p* = 0.005) participants had high risk AUD compared to single ones, whereas a significantly higher percentage of widowed (84.2% vs 47.2%; *p* = 0.002) and divorced (73.3% vs 47.2%l *p* = 0.007) participants had high risk AUD compared to married ones.

**Table 1:** Sociodemographic characteristics of the sample population

	Alcohol use disorder		*p*-value
	Low risk	High risk	
	Frequency (%)	Frequency (%)	
**Gender**			
**Male**	173 (41.2%)	247 (58.8%)	**< 0.001**
**Female**	216 (61.9%)	133 (38.1%)	
**Education level**			
**Illiterate**	4 (33.3%)	8 (66.7%)	**< 0.001**
**Primary**	9 (23.1%)	30 (76.9%)	
**Complementary**	18 (34.6%)	34 (65.4%)	
**Secondary**	57 (51.8%)	53 (48.2%)	
**University**	258 (55.8%)	204 (44.2%)	
**higher education**	36 (56.3%)	28 (43.8%)	
**Socioeconomic status**			
**< 1000 $**	195 (51.9%)	181(48.1%)	0.446
**1000–2000 $**	126 (48.8%)	132 (51.2%)	
**> 2000 $**	47 (45.2%)	57 (54.8%)	
**Marital status**			
**Single**	257 (52.9%)	229 (47.1%)	**0.001**
**Married**	124 (52.8%)	111 (47.2%)	
**Widowed**	3 (15.8%)	16 (84.2%)	
**Divorced**	8 (26.7%)	22 (73.3%)	
	**Mean ± SD**	**Mean ± SD**	
**Age (in years)**	30.27 ± 13.14	30.33 ± 11.90	0.945
**Number of kids**	0.91 ± 1.62	0.62 ± 1.28	**0.008**

Bold numbers indicate significant *p*-values

### Bivariate analysis

Higher alexithymia (54.55 vs. 49.73; *p* < 0.001), depression (17.31 vs. 8.00; *p* < 0.001), anxiety (17.58 vs. 10.90; *p* < 0.001), perceived stress (19.37 vs. 17.64; *p* < 0.001), emotional work fatigue (20.32 vs. 15.29; *p* < 0.001), physical work fatigue (18.57 vs. 17.52; p < 0.001), mental work fatigue (17.63 vs 14.05; *p* < 0.001) and suicidal ideation (1.00 vs. 0.17; *p* = 0.001) were significantly found in patients at high risk AUD compared to those at low risk. Moreover, higher emotional awareness, emotional management, social emotional awareness and relationship management scores were found in patients with low risk AUD compared to those at high risk (Table 2).

**Table 2:** Association between the alcohol use disorder scale score and all other scales

	Alcohol dependence		*p*-value
	Low risk	High risk	
	Mean ± SD	Mean ± SD	
Alexithymia Scale (TAS-20)	49.73 ± 10.01	54.55 ± 10.30	< 0.001
Depression score (HAM-D)	8.00 ± 7.93	17.31 ± 10.71	< 0.001
Anxiety score (HAM-A)	10.90 ± 9.40	17.58 ± 9.69	< 0.001
Perceived stress scale (PSC)	17.64 ± 6.14	19.37 ± 5.58	< 0.001
Liebowitz social anxiety scale	36.58 ± 24.77	42.75 ± 20.59	< 0.001
Emotional awareness	20.32 ± 7.71	17.68 ± 7.00	0.097
Emotional management	22.25 ± 8.82	17.54 ± 7.13	< 0.001
Social Emotional awareness	23.81 ± 8.80	19.59 ± 7.64	0.005
Relationship management	23.69 ± 9.15	19.35 ± 7.87	< 0.001
Emotional work fatigue	15.29 ± 9.73	20.32 ± 11.12	< 0.001
Physical work fatigue	17.52 ± 8.19	18.57 ± 8.36	< 0.001
Mental work fatigue	14.05 ± 7.97	17.63 ± 9.51	< 0.001
Suicidal ideation score	0.17 ± 0.73	1.00 ± 1.50	0.001

### Factor analysis

All items of the study instrument could be extracted and used in the analyses. The total items converged over a solution of 4 factors: Factor one indicates mental wellbeing (i.e. low emotional work fatigue and high emotional intelligence); Factor two indicates psychological distress (i.e. high mental and physical work fatigue, high stress and alexithymia); Factor three indicates mood/affective dysfunction (i.e. high suicidal ideation, high depression and high anxiety); Factor four indicates social dysfunction (i.e. low self-esteem and high social phobia) explaining a total of 65.61% of the variance (KMO = 0.832; Bartlett’s test of sphericity *p* < 0.001) (Table 3).

**Table 3:** Pattern loading of the major factor solutions after promax rotation, without taking the alcohol use disorders (AUDIT score) among these factors

	Factor 1	Factor 2	Factor 3	Factor 4
High social emotional awareness	0.881			
High relationship management	0.868			
High emotional management	0.831			
High emotional awareness	0.813			
Low emotional work fatigue	0.706			
High physical work fatigue		0.826		
High perceived stress		0.814		
High alexithymia Scale (TAS-20)		0.744		
High mental work fatigue		0.594		
High suicidal ideation			0.867	
High depression score (HAM-D)			0.833	
High anxiety score (HAM-A)			0.658	
Low Rosenberg self-esteem				0.863
High Liebowitz total score				0.452

Factor 1 = mental wellbeing (i.e. high emotional intelligence and low emotional work fatigue; Factor 2 = psychological distress (i.e. high physical and mental work fatigue, high stress and high alexithymia; Factor 3 = mood/affective dysfunction (i.e. high suicidal ideation, high depression and high anxiety; Factor 4 = social dysfunction (i.e. low self-esteem and high social phobia)

### Profiles of participants

Based on the 4 factors, a cluster analysis derived three mutually exclusive clusters. Cluster number one formed 45.4% of the sample and assembled people with psychological difficulties (work fatigue and high stress, high emotional work fatigue and low emotional intelligence, low self-esteem, high social phobia, high alexithymia); Cluster number two formed 34.4% of the sample and assembled people with high wellbeing (low suicidal ideation, low emotional work fatigue, depression and anxiety, high emotional intelligence, high self-esteem and low social phobia); whereas cluster 3 formed 20.2% of the sample and represented people with mental dysfunction (high anxiety and depression, high suicidal ideation, low self-esteem and high social phobia, low emotional intelligence, high emotional work fatigue) (Table 4).

**Table 4:** Classification of participants in the study sample by cluster analysis using the categories factor scoring

	Cluster 1 *N* = 269 (45.4%)	Cluster 2 *N* = 204 (34.4%)	Cluster 3 *N* = 120 (20.2%)
**Factor 1:** High emotional intelligence & low emotional work fatigue	−0.34	0.92	−0.79
**Factor 2:** High physical and mental work fatigue, high stress & high alexithymia	0.53	−0.57	−0.23
**Factor 3:** High suicidal ideation & high depression and anxiety	0.31	−0.86	0.76
**Factor 4:** low self-esteem & high social phobia.	0.71	−0.42	−0.87

Cluster 1 = **People with psychological difficulties** (low self-esteem, high social phobia, high alexithymia, high physical and mental work fatigue and high stress, low emotional intelligence and high emotional work fatigue); cluster 2 = **People with high wellbeing (**high emotional intelligence and low emotional work fatigue, with low suicidal ideation, low depression and anxiety, high self-esteem and low social phobia); cluster 3 = **People in distress (**High suicidal ideation, high depression and anxiety, with low self-esteem & high social phobia)

### Multivariable analyses

The results of a first backward logistic regression, taking the dichotomous AUDIT variable as the dependent variable and the sociodemographic characteristics as independent variables, showed that being divorced (ORa = 6.723; CI 1.379–32.784; *p* = 0.018) was correlated with higher AUD risk in a significant way. Being a female (ORa = 0.431; CI 0.308–0.605; *p < 0.001*), having a higher number of kids (ORa = 0.656; CI 0.526–0.819; *p < 0.001*) and a high university degree (ORa = 0.204; CI 0.048–0.860; *p* = 0.03) were significantly associated with lower AUD risk.

The multivariable analyses results in models 2 to 5 were adjusted for the sociodemographic characteristics. A second backward logistic regression, taking the dichotomous AUDIT variable as the dependent variable, showed that higher alexithymia (ORa = 1.030; CI 1.009–1.051; *p* = 0.004), depression (ORa = 1.076; CI 1.050–1.103; *p < 0.001*) and suicidal ideation (ORa = 1.253; CI 1.026–1.531; *p* = 0.027) were significantly associated with higher AUD risk. Finally, higher emotional management (ORa = 0.962; CI 0.937–0.988; *p* = 0.005) was significantly associated with lower AUD risk.

A third linear regression, taking the continuous AUDIT score as the dependent variable, showed that depression (Beta = 0.282; CI 0.220–0.344; *p < 0.001*), alexithymia (Beta = 0.146; CI 0.093–0.200; *p < 0.001*) and suicidal ideation (Beta = 0.855; CI 0.385–1.325; *p < 0.001*) were linked to higher AUDIT scores, while higher emotional management (Beta = − 0.079; CI -0.150- -0.008; *p* = 0.03) was associated with lower AUDIT scores.

Fourth linear regression, taking the continuous AUDIT score as the dependent variable and the factors obtained in the factor analysis as independent variables, showed that Factor 2 (Psychological distress) (Beta = 1.107; CI 0.496–1.719; *p < 0.001*) and Factor 3 (mood/affective dysfunction) (Beta = 3.330; CI 2.672–3.989; *p < 0.001*) were associated with higher AUDIT scores, whereas Factor 1 (mental wellbeing) (Beta = − 0.817; CI -1.417- -0.217; *p* = 0.008), was associated with lower AUDIT scores.

The fifth linear regression was obtained with the continuous AUDIT score chosen as the dependent variable while the obtained clusters were taken as independent variables. The regression showed that people with psychological difficulties (cluster 1) (Beta = 5.547; CI 4.430–6.663; *p < 0.001*) and people in distress (cluster 3) (Beta = 7.455; CI 5.945–8.965; *p < 0.001*) were associated with higher AUDIT scores compared to participants with high wellbeing (cluster 2) (Table 5).

**Table 5:** Multivariable analysis

Model 1: Logistic regression taking the dichotomous alcohol use disorder scale score (low vs. high risk) as the dependent variable and the sociodemographic characteristics as independent variables.					
	OR	*p*-value	Confidence interval		
			Lower Bound	Lower Bound	
Gender (females vs males^a^)	0.431	< 0.001	0.308	0.605	
Divorced vs single^a^	6.723	0.018	1.379	32.784	
Number of kids	0.656	< 0.001	0.526	0.819	
Secondary education level vs illiterate^a^	0.272	0.083	0.062	1.185	
University education level vs illiterate^a^	0.204	0.030	0.048	0.860	
Variables entered: Gender, Marital status, number of kids, education level					
Model 2: Logistic regression taking the dichotomous alcohol use disorder scale score (low vs. high risk) as the dependent variable.					
Gender (females vs males^a^)	0.460		< 0.001	0.305	0.694
Alexithymia Scale (TAS-20)	1.030		0.004	1.009	1.051
Depression score (HAM-D)	1.076		< 0.001	1.050	1.103
Emotional management	0.962		0.005	0.937	0.988
Suicidal ideation score	1.253		0.027	1.026	1.531
Number of kids	0.863		0.037	0.752	0.991
Variables entered: Gender, Marital status, number of kids, education level, TAS_20, HAMD score, HAMA score, PSC score, Liebowitz score, Emotional awareness score, Emotional management score, Social emotional awareness score, Relationship management score, MBI - Emotional exhaustion, MBI - Personal accomplishment, MBI - Depersonalization, Suicidal ideation score.					
Model 3: Linear regression taking the continuous AUDIT score as the dependent variable and all the scales as independent variables.					
	Unstandardized Beta	Standardized Beta	*p*-value	Confidence interval	
				Lower Bound	Upper Bound
Depression score (HAM-D)	0.282	0.354	< 0.001	0.220	0.344
Alexithymia Scale (TAS-20)	0.146	0.189	< 0.001	0.093	0.200
Suicidal ideation score	0.855	0.134	< 0.001	0.385	1.325
Emotional management	−0.079	−0.078	0.030	−0.150	−0.008
Gender (females vs males^a^)	−2.647	−0.160	< 0.001	−3.771	−1.523
Secondary education vs illiterate^a^	−2.476	−0.103	0.012	−4.415	−0.538
University education vs illiterate^a^	− 2.579	−0.148	< 0.001	−4.024	− 1.134
Intermediate vs low^a^ SES	1.167	0.067	0.050	0.001	2.333
Variables entered: Age, Gender, SES, education level, TAS_20, HAMD score, HAMA score, PSC score, Liebowitz score, Emotional awareness score, Emotional management score, Social emotional awareness score, Relationship management score, MBI - Emotional exhaustion, MBI - Personal accomplishment, MBI - Depersonalization, Suicidal ideation score. ^a^SES = socioeconomic status (Reference = low socioeconomic status).					
Model 4: Linear regression taking the continuous AUDIT score as the dependent variable and four factors obtained in the factor analysis as independent variables.					
Mental Wellbeing (Factor 1)	−0.817	−0.099	0.008	−1.417	−0.217
Psychological distress (Factor 2)	1.107	0.136	< 0.001	0.496	1.719
Mood/affective dysfunction (Factor 3)	3.330	0.398	< 0.001	2.672	3.989
Gender (females vs males^a^)	−2.613	−0.158	< 0.001	−3.764	−1.462
Secondary education vs illiterate^a^	−2.505	−0.105	0.014	−4.507	−0.503
University education vs illiterate^a^	−2.678	−0.153	< 0.001	−4.165	−1.190
Factor 1 = mental wellbeing (i.e. high emotional intelligence and low emotional work fatigue; Factor 2 = psychological distress (i.e. high physical and mental work fatigue, high stress and high alexithymia; Factor 3 = mood/affective dysfunction (i.e. high suicidal ideation, high depression and high anxiety; Factor 4 = social dysfunction (i.e. low self-esteem and high social phobia). Variables entered in the model: Factor 1, Factor 2, Factor 3, factor 4, Age, Gender, SES, education level.					
Model 5: Linear regression taking the continuous AUDIT score as the dependent variable and the three clusters as independent variables.					
People with psychological difficulties (Cluster 1)	5.547	0.325	< 0.001	4.430	6.663
People in distress (Cluster 3)	7.455	0.323	< 0.001	5.945	8.965
Gender (Male^a^ vs. Female)	−3.011	−0.184	< 0.001	−4.036	−1.985
Secondary education vs illiterate^a^	−2.461	−0.104	0.008	− 4.265	−.657
University education vs illiterate^a^	−3.045	−0.175	< 0.001	−4.392	−1.698
Divorced vs. single^a^	5.047	0.118	< 0.001	2.397	7.698
Widowed vs. single^a^	4.962	0.091	0.004	1.545	8.379
Variables entered in the model: cluster 1, cluster 2, cluster 3, Age, Gender, SES, education level Cluster 1 = **People with psychological difficulties** (low self-esteem, high social phobia, high alexithymia, high physical and mental work fatigue and high stress, low emotional intelligence and high emotional work fatigue); cluster 2 = **People with high wellbeing (**high emotional intelligence and low emotional work fatigue, with low suicidal ideation, low depression and anxiety, high self-esteem and low social phobia); cluster 3 = **People in distress (**High suicidal ideation, high depression and anxiety, with low self-esteem & high social phobia).					
^a^Reference group					

The results of the logistic regressions using the ENTER method for all models can be found in Supplementary Table 1.

## Discussion

In Lebanon, there have been no studies that aimed to evaluate factors associated with alcohol use disorder among its population.

### Independent factors associated with AUD

Our results showed that work fatigue, as indicated by high scores of physical and mental fatigue at work, was associated with a higher risk of alcohol use disorder, consistent with previous studies [52–54]. Psychosocial stress at work, characterized by an imbalance between efforts spent in terms of psychological and physical load and reward (such as money, esteem and career opportunities) [55], is a risk factor for alcohol use disorder [56]. In fact, heavy workload or demanding jobs make workers with little personal and social resources prone to both work fatigue and addictive behavior. At the neurobiological level, work fatigue and alcohol use disorder are associated because of the link between stress and alcohol consumption [57]. Alcohol appears to be a stress reliever when craving for stress alleviation, this process involves both intracellular and dopaminergic extracellular mechanisms [57].

Our results also showed high alcohol consumption in response to a higher number of stressors. Alcoholism, alcohol abuse, heavy drinking and alcohol dependence were previously shown to be directly related to stressful living situations and persistent stressors [58]. However, while some studies have reported positive associations, others have found negative associations [59, 60]. Alcohol is an effective anxiolytic and serves as a coping strategy against stress and work fatigue [61] .

Regarding the sociodemographic characteristics and their relationship with AUD, married individuals had lower AUDIT scores. This may be due to the tight connections among each family and solidarity between different generations, which is one of the fundamental qualities of Middle Eastern/Arab countries. The executed research also found that female gender was associated with lower AUDIT scores. As previous studies showed, men were more prone to alcoholism than women [62–64]. This could be related to the cultural or religious norms and values that make women less comfortable reporting embarrassing or prohibited behaviors.

### Factors associated with AUD

Our results demonstrated a negative relationship between emotional intelligence, emotional work fatigue (Factor 1) and alcohol use disorders. One possible hypothesis is that when individuals are more capable of managing and controlling their own emotions, and to a certain extent the emotions of others, they would be less likely to report feeling alienated and distant from others, and therefore do not have addictive behaviors such as alcohol. To our knowledge, no studies have revealed the correlation between these factors at the same time. Based on our findings, emotional intelligence was lower in participants with high AUD risk compared to those at low risk, consistent with previous studies [65–67]. There is evidence that those who become addicted to alcohol are not able to understand and talk about their feelings [66]. They use alcohol to relax and remove their ambiguous and unknown stresses and discomforts.

Our results showed that psychological difficulties (Factor 2), including high alexithymia, high stress, high physical and mental work fatigue, were associated with higher AUD risk. A higher alexithymia score, as shown by a higher TAS score, was associated with a higher AUD risk. Our study corroborates the findings of a previous study where higher self-reporting of alcohol and nicotine cravings and stress were associated with difficulties in recognizing and expressing emotions [68]. Furthermore, alcohol helps relieve stressful conditions and improves interpersonal activity in people with alexithymia [69]. These people consume alcohol to become more outgoing, sociable and self-assured, and find it easier to express their feeling after alcohol consumption [19]. Individuals with a high alexithymia, and higher overall work fatigue scores [70] are often associated with low work-related interest, complications in interpersonal relationships, and a poor physical situation, with higher score of perceived stress, and use alcohol as an escape from stress and confused emotions [71].

Positive and significant correlation was shown between mood/affective disorders (Factor 3: high suicidal thoughts, high anxiety and depression) and higher AUDIT scores. Alcoholism can have negative effects on mental status, and is often associated with mood disorders [72]. In fact, AUD can intensify impulsivity and aggravate those symptoms by stimulating the thoughts of hopelessness and sadness. In a recent review, the reciprocal association was demonstrated, with mood disorders being precursors of alcoholism [1]. Actually, this comorbidity can be a cyclic process [73, 74]. According to Watts, most individuals with mood disorders abuse alcohol in search of pleasure and disinhibition or for the reduction of cognitive, emotional and behavioral symptoms of depression [75]. However, the state of intoxication induced by alcohol abuse can increase impulsivity and promote feelings and thoughts of sadness, thus, worsening mood disorder symptoms [75]. Similarly, anxiety was prevalent in people with AUD. Dangerous alcohol use, refusal or increased dropout rates of AUD treatment was associated with a concurrent diagnosis of anxiety [76, 77]. Furthermore, our results confirm that high rates of suicide are associated with higher AUDIT scores. The results of a meta-analysis demonstrated that the risk of suicidal ideation, suicide attempts, and suicide was increased by 2–3 times by AUD [78], which is considered a significant predictor of suicide [78].

Besides, the attained data showed an interconnection between social dysfunction (Factor 4; low self-esteem and high social phobia) and high risk of AUD. Self-esteem plays a major role in social anxiety disorder [79]; while lowered self-esteem may put the individual at risk of later social anxiety, having an anxiety disorder can also make him feel worse about himself. Controversial results were found between social anxiety and alcohol use, with some findings showing a positive association [80–83], while others showed no relationship [84]. One predominant mechanism suggested that lower levels of tension and stress are the main link between social anxiety, self-esteem and alcohol use [85]. According to this model (tension reduction hypothesis), alcohol acts as a negative reinforcer to reduce social stress and anxiety [84, 86].

Finally, the sample was categorized into three groups/clusters. The present results were reasonable since Lebanese are vulnerable to mental disorders for many reasons: a study conducted in 2003 has found that approximatively 50% of the population in Lebanon was traumatized due to the war [87]. The unstable political condition, the history of wars and terrorist attacks [88] and the absence of a clean environment [89] in Lebanon would consequently contribute to the increase in mental disorders among this population. Another contributor is the increased flow of Syrian refugees that decreased employment rates [89] and xenophobic mindsets among the Lebanese [90]. This is concordant with how the public eye looks at mental illness as a mark of disgrace [91], and the undeclared forbiddance to seek a treatment from a healthcare professional for such disorders [92].

#### Clinical implications

Alcohol is a psychoactive substance, legally produced and sold, the production and marketing of which can be easily controlled and regulated by local authorities through the serious implementation of laws and policies. Further, recognition of risk factors is the starting point for identifying and preventing AUD among vulnerable people and for treating symptoms already experienced. It is essential to bring attention towards factors associated with alcohol use disorder and prevent/treat them.

#### Limitations

Our study is cross-sectional, therefore, causation can’t be inferred. Some scales translated into the Arabic language have not been validated yet. Recall bias might be present since the study is retrospective and can lead to the overestimation of effects for some known factors recognized to be associated with alcohol use disorder. Participants might have misapprehended few questions, symptoms may have been either over/under evaluated. These two inconveniences might present a major source of information bias. The refusal rate is also a main reason behind the selection bias.

## Conclusion

AUD seems to be influenced by several factors among the Lebanese population, including stress, anxiety and work fatigue. Healthcare professionals should spread awareness to reduce the prevalence of these factors. Screening and periodic monitoring of alcohol consumption are recommended, as well as therapeutic intervention among the population.

## Supplementary information

**Additional file 1.** Supplementary Table 1: Multivariable analysis using the ENTER method for all models.

## Data Availability

All data generated or analyzed during this study are not publicly available to maintain the privacy of the individuals’ identities. The dataset supporting the conclusions is available upon request to the corresponding author.

## Abbreviations

3D-WFI: Evaluation of the Three-Dimensional Work Fatigue Inventory
AUDIT: Alcohol Use Disorders Identification Test
C-SSRS: Columbia–Suicide Severity Rating Scale
HAD: Hazardous Alcohol Drinking
HAM-A: Hamilton anxiety scale
HDRS: Hamilton depression rating scale
KMO: Kaiser–Meyer–Olkin
LSAS: Liebowitz Social Anxiety Scale
PSS: Perceived Stress Scale
RSES: Rosenberg self-esteem scale
SES: Socioeconomic status
TAS: Toronto Alexithymia Scale
WHO: World Health Organization
