# Supplementary material for: Factors associated with alcohol use disorder: the role of depression, anxiety, stress, alexithymia and work fatigue- a population study in Lebanon
Source: BMC Public Health. 2020 Feb 18;20:245. doi: 10.1186/s12889-020-8345-1 (PMC7029557; doi:10.1186/s12889-020-8345-1)
Supplement: Supplementary file 1 — Additional file 1. Supplementary Table 1: Multivariable analysis using the ENTER method for all models. [file 12889_2020_8345_MOESM1_ESM.docx]

| **Supplementary Table 1: Multivariable analysis using the ENTER method for all models.** | | | | | | | | | | |
| --- | --- | --- | --- | --- | --- | --- | --- | --- | --- | --- |
| **Model 1: Logistic regression taking the dichotomous alcohol use disorder scale score (high vs low risk) as the dependent variable and the sociodemographic characteristics as independent variables.** | | | | | | | | | | |
|  | | **OR** | | | **p-value** | | **Confidence interval** | | | |
|  |  |  |  |  |  |  | **Lower Bound** | | **Lower Bound** | |
| **Gender (females vs males*)** | | 0.429 | | | **<0.001** | | 0.307 | | 0.601 | |
| **Education level** | |  | | | **<0.001** | |  | |  | |
| Illiterate | | 1 | | |  | |  | |  | |
| Primary | | 1.723 | | | 0.524 | | 0.323 | | 9.186 | |
| Complementary | | 0.457 | | | 0.331 | | 0.094 | | 2.215 | |
| Secondary | | 0.271 | | | 0.085 | | 0.062 | | 1.197 | |
| University | | 0.207 | | | **0.034** | | 0.048 | | 0.886 | |
| Higher university | | 0.119 | | | **0.009** | | 0.024 | | 0.586 | |
| **Marital status** | |  | | | **0.02** | |  | |  | |
| Single | | 1 | | |  | |  | |  | |
| Married | | 1.742 | | | 0.115 | | 0.873 | | 3.475 | |
| Widowed | | 6.825 | | | **0.015** | | 1.471 | | 35.595 | |
| Divorced | | 0.631 | | | **<0.001** | | 0.504 | | 0.790 | |
| **Number of kids** | | 0.631 | | | **<0.001** | | 0.504 | | 0.790 | |
| Variables entered: Gender, Marital status, number of kids, education level | | | | | | | | | | |
| **Model 2: Logistic regression taking the dichotomous alcohol use disorder scale score (high vs low risk) as the dependent variable.** | | | | | | | | | | |
|  | | | **OR** | | | **p-value** | | **Confidence interval** | | |
|  |  |  |  |  |  |  |  | **Lower Bound** | | **Lower Bound** |
| **Gender (females vs males*)** | | | 0.486 | | | **0.001** | | 0.318 | | 0.741 |
| **Marital status** | | |  | | | 0.511 | |  | |  |
| Single | | | 1 | | |  | |  | |  |
| Married | | | 1.391 | | | 0.406 | | 0.638 | | 3.033 |
| Widowed | | | 2.581 | | | 0.556 | | 0.110 | | 60.802 |
| Divorced | | | 3.171 | | | 0.205 | | 0.532 | | 18.888 |
| **Number of kids** | | | 0.803 | | | 0.072 | | 0.632 | | 1.020 |
| **Alexithymia Scale** | | | 1.031 | | | **0.01** | | 1.007 | | 1.055 |
| **Depression score** | | | 1.076 | | | **<0.001** | | 1.044 | | 1.109 |
| **Anxiety score** | | | 0.988 | | | 0.435 | | 0.960 | | 1.018 |
| **Stress** | | | 0.986 | | | 0.509 | | 0.946 | | 1.028 |
| **Social phobia total score** | | | 0.997 | | | 0.574 | | 0.987 | | 1.007 |
| **Emotional awareness** | | | 1.020 | | | 0.360 | | 0.978 | | 1.063 |
| **Emotional management** | | | 0.959 | | | **0.04** | | 0.921 | | 0.998 |
| **Social emotional awareness** | | | 0.996 | | | 0.859 | | 0.953 | | 1.041 |
| **Relationship management** | | | 0.986 | | | 0.503 | | 0.944 | | 1.028 |
| **Emotional work fatigue** | | | 1.013 | | | 0.378 | | 0.985 | | 1.041 |
| **Mental work fatigue** | | | 0.999 | | | 0.938 | | 0.965 | | 1.033 |
| **Physical work fatigue** | | | 1.004 | | | 0.817 | | 0.973 | | 1.036 |
| **Suicidal ideation score** | | | 1.379 | | | **0.013** | | 1.069 | | 1.778 |
| Variables entered: Gender, Marital status, number of kids, education level, TAS_20, HAMD score, HAMA score, PSC score, Liebowitz score, Emotional awareness score, Emotional management score, Social emotional awareness score, Relationship management score, MBI - Emotional exhaustion, MBI - Personal accomplishment, MBI - Depersonalization, Suicidal ideation score. | | | | | | | | | | |
| **Model 3: Linear regression taking the continuous AUDIT score as the dependent variable and all the scales as independent variables.** | | | | | | | | | | |
|  | **Unstandardized Beta** | | | **Standardized Beta** | | **p-value** | | **Confidence interval** | | |
|  |  |  |  |  |  |  |  | **Lower Bound** | | **Upper Bound** |
| **Age** | 0.028 | | | 0.041 | | 0.381 | | -0.035 | | 0.092 |
| **Alexithymia** | 0.141 | | | 0.182 | | **<0.001** | | 0.078 | | 0.204 |
| **Depression score** | 0.250 | | | 0.313 | | **<0.001** | | 0.173 | | 0.327 |
| **Anxiety score** | -0.007 | | | -0.008 | | 0.866 | | -0.088 | | 0.074 |
| **Stress score** | -0.051 | | | -0.039 | | 0.363 | | -0.161 | | 0.059 |
| **Social phobia total score** | 0.002 | | | 0.004 | | 0.907 | | -0.026 | | 0.029 |
| **Emotional awareness** | 0.060 | | | 0.051 | | 0.278 | | -0.049 | | 0.169 |
| **Emotional management** | -0.075 | | | -0.074 | | 0.164 | | -0.180 | | 0.031 |
| **Social emotional awareness** | -0.065 | | | -0.066 | | 0.279 | | -0.183 | | 0.053 |
| **Relationship management** | -0.012 | | | -0.012 | | 0.842 | | -0.126 | | 0.103 |
| **Emotional work fatigue** | 0.015 | | | 0.019 | | 0.700 | | -0.062 | | 0.092 |
| **Mental work fatigue** | 0.049 | | | 0.048 | | 0.293 | | -0.042 | | 0.141 |
| **Physical work fatigue** | 0.012 | | | 0.013 | | 0.778 | | -0.074 | | 0.099 |
| **Suicidal ideation** | 1.195 | | | 0.172 | | **<0.001** | | 0.63 | | 1.737 |
| **Gender (females vs males*)** | -2.438 | | | -0.147 | | **<0.001** | | -3.598 | | -1.278 |
| **Intermediate vs low* monthly income** | 1.186 | | | 0.068 | | 0.077 | | -0.130 | | 2.501 |
| **High vs low* monthly income** | 0.672 | | | 0.029 | | 0.482 | | -1.206 | | 2.550 |
| **Married vs single*** | -1.125 | | | -0.062 | | 0.162 | | -2.703 | | 0.453 |
| **Widowed vs single*** | -0.071 | | | -0.001 | | 0.972 | | -3.999 | | 3.856 |
| **Divorced vs single*** | 0.905 | | | 0.022 | | 0.549 | | -2.058 | | 3.867 |
| **Primary education vs illiterate*** | 1.286 | | | 0.034 | | 0.435 | | -1.947 | | 4.519 |
| **Complementary education vs illiterate*** | 0.156 | | | 0.005 | | 0.917 | | -2.798 | | 3.110 |
| **Secondary education vs illiterate*** | -2.095 | | | -0.087 | | 0.099 | | -4.588 | | 0.397 |
| **University education vs illiterate*** | -2.250 | | | -0.129 | | **0.042** | | -4.417 | | -0.083 |
| Variables entered: Age, Gender, SES, education level, TAS_20, HAMD score, HAMA score, PSC score, Liebowitz score, Emotional awareness score, Emotional management score, Social emotional awareness score, Relationship management score, MBI - Emotional exhaustion, MBI - Personal accomplishment, MBI - Depersonalization, Suicidal ideation score.  *SES= socioeconomic status (Reference= low socioeconomic status). | | | | | | | | | | |
| **Model 4: Linear regression taking the continuous AUDIT score as the dependent variable and four factors obtained in the factor analysis as independent variables.** | | | | | | | | | | |
|  | **Unstandardized Beta** | | | **Standardized Beta** | | **p-value** | | **Confidence interval** | | |
|  |  |  |  |  |  |  |  | **Lower Bound** | | **Upper Bound** |
| **Age** | 0020 | | | 0.029 | | 0.455 | | -0.033 | | 0.073 |
| **Gender (females vs males*)** | -1.407 | | | -0.086 | | **0.004** | | -2.365 | | -0.449 |
| **Intermediate vs low* monthly income** | 1.236 | | | 0.071 | | **0.025** | | 0.153 | | 2.319 |
| **High vs low* monthly income** | 0.963 | | | 0.040 | | 0.229 | | -0.607 | | 2.534 |
| **Married vs single*** | -0.763 | | | -0.042 | | 0.250 | | -2.066 | | 0.539 |
| **Widowed vs single*** | -0.768 | | | -0.014 | | 0.647 | | -4.061 | | 2.524 |
| **Divorced vs single*** | 1.398 | | | 0.033 | | 0.276 | | -1.119 | | 3.915 |
| **Primary education vs illiterate*** | 0.592 | | | 0.015 | | 0.670 | | -2.134 | | 3.318 |
| **Complementary education vs illiterate*** | -0.353 | | | -0.011 | | 0.775 | | -2.775 | | 2.070 |
| **Secondary education vs illiterate*** | -2.325 | | | -0.099 | | **0.029** | | -4.415 | | -0.235 |
| **University education vs illiterate*** | -2.314 | | | -0.134 | | **0.013** | | -4.138 | | -0.489 |
| **Mental Wellbeing (Factor 1)** | -1.405 | | | -0.172 | | **<0.001** | | -1.879 | | -0.931 |
| **Psychological distress (Factor 2)** | 2.133 | | | 0.264 | | **<0.001** | | 1.661 | | 2.605 |
| **Mood/affective dysfunction (Factor 3)** | 4.213 | | | 0.511 | | **<0.001** | | 3.723 | | 4.703 |
| Factor 1= mental wellbeing (i.e. high emotional intelligence and low emotional work fatigue; Factor 2= psychological distress (i.e. high physical and mental work fatigue, high stress and high alexithymia; Factor 3= mood/affective dysfunction (i.e. high suicidal ideation, high depression and high anxiety; Factor 4= social dysfunction (i.e. low self-esteem and high social phobia).  Variables entered in the model: Factor 1, Factor 2, Factor 3, factor 4, Age, Gender, SES, education level. | | | | | | | | | | |
| **Model 5: Linear regression taking the continuous AUDIT score as the dependent variable and the three clusters as independent variables.** | | | | | | | | | | |
|  | **Unstandardized Beta** | | | **Standardized Beta** | | **p-value** | | **Confidence interval** | | |
|  |  |  |  |  |  |  |  | **Lower Bound** | | **Upper Bound** |
| **Age** | -0.040 | | | -0.06 | | 0.083 | | -0.085 | | 0.005 |
| **Gender (females vs males*)** | -1.902 | | | -0.117 | | **<0.001** | | -2.727 | | -1.077 |
| **Intermediate vs low* monthly income** | 0.697 | | | 0.040 | | 0.153 | | -0.258 | | 1.652 |
| **High vs low* monthly income** | 0.362 | | | 0.015 | | 0.600 | | -0.990 | | 1.714 |
| **Married vs single*** | 0.059 | | | 0.003 | | 0.920 | | -1.090 | | 1.208 |
| **Widowed vs single*** | 2.496 | | | 0.046 | | 0.081 | | -0.305 | | 5.298 |
| **Divorced vs single*** | 3.711 | | | 0.087 | | **0.001** | | 1.486 | | 5.936 |
| **Primary education vs illiterate*** | -0.258 | | | -0.007 | | 0.830 | | -2614 | | 2.098 |
| **Complementary education vs illiterate*** | -1.495 | | | -0.046 | | 0.175 | | -3.657 | | 0.666 |
| **Secondary education vs illiterate*** | -2.206 | | | -0.094 | | **0.02** | | -4.063 | | -0.348 |
| **University education vs illiterate*** | -2.731 | | | -0.157 | | **0.001** | | -4.376 | | -1.087 |
| **People with psychological difficulties (Cluster 1)** | 1.715 | | | 0.095 | | **0.001** | | 0.742 | | 2.688 |
| **People in distress (Cluster 3)** | 12.401 | | | 0.702 | | **<0.001** | | 11.413 | | 13.390 |
| Variables entered in the model: cluster 1, cluster 2, cluster 3, Age, Gender, SES, education level  Cluster 1= **People with psychological difficulties** (low self-esteem, high social phobia, high alexithymia, high physical and mental work fatigue and high stress, low emotional intelligence and high emotional work fatigue); cluster 2= **People with high wellbeing (**high emotional intelligence and low emotional work fatigue, with low suicidal ideation, low depression and anxiety, high self-esteem and low social phobia); cluster 3= **People in distress (**High suicidal ideation, high depression and anxiety, with low self-esteem & high social phobia). | | | | | | | | | | |
| *Reference group; Numbers in bold indicate significant p-values. | | | | | | | | | | |
